# Revisit the Effectiveness of Educational Kinesiology on Stress and Anxiety Amelioration in Kindergarteners With Special Needs Using Biological Measures

**DOI:** 10.3389/fpsyt.2021.773659

**Published:** 2021-12-10

**Authors:** Alan Pui-Lun Tai, Way Kwok-Wai Lau

**Affiliations:** ^1^Department of Special Education and Counselling, The Education University of Hong Kong, Hong Kong, Hong Kong SAR, China; ^2^Integrated Centre for Wellbeing, The Education University of Hong Kong, Hong Kong, Hong Kong SAR, China; ^3^Bioanalytical Laboratory for Educational Sciences, The Education University of Hong Kong, Hong Kong, Hong Kong SAR, China

**Keywords:** anxiety, educational kinesiology, kindergarten, special needs, stress, cortisol, oxytocin

## Abstract

**Background:** Educational kinesiology is a popular intervention that aims to improve brain functioning via physical movements. Yet, it lacks supporting scientific evidence and is regarded as pseudoscience. Given the popularity of educational kinesiology in school settings, it is important to revisit its effectiveness through scientific research. Previous studies that evaluated the effectiveness of educational kinesiology relied mainly on subjective measures, in which subjective bias is inevitable. Cortisol and oxytocin levels in saliva have been reported to be reliable stress and anxiety markers that provide unbiased objective data. This study explores the effect of educational kinesiology on the changes in salivary cortisol and oxytocin levels in kindergarteners with special needs.

**Methods:** A quasi-experimental design was adopted in this study. Thirty-seven kindergarteners (3.5–6.5 years old) who were either diagnosed with one type of special needs or referred by school principals due to the requirement of special supports at school were assigned to either the intervention group, which received 1-h educational kinesiology intervention weekly for a total of 10 weeks, or the wait-list control group. Saliva samples were collected at baseline and after the completion of intervention programme for the measurement of cortisol and oxytocin levels. Scores of Parent-rated Preschool Anxiety Scale (PAS-TC) were also collected at pre- and post-intervention. Because of the small samples, non-parametric tests such as Mann-Whitney U test, Quade test, and Fisher's exact tests were used in this study where appropriate.

**Results:** After controlled for the effect at baseline, gender and types of special needs, the changes in oxytocin levels were significantly higher in the intervention group compared with control [*F*_(1, 35)_ = 4.747, *p* = 0.036, eta^2^ = 0.119], whereas no significant between-group difference in changes of cortisol levels was observed [*F*_(1, 35)_ = 0.306, *p* = 0.584, eta^2^ = 0.009]. Results from PAS-TC showed significant improvement in anxiety levels after the intervention in the intervention group (*p* = 0.048, ϕ = 0.344, *p* = 0.037).

**Conclusions:** Our findings suggest a plausible anti-anxiety effect of educational kinesiology in kindergarteners with special needs by elevating the oxytocin levels. Future studies are warranted to further confirm our findings with a larger sample.

## Introduction

Depression and anxiety are common mental health problems that significantly affect children in the modern society. In western countries, up to 14% of preschool-age children were found to have clinical levels of depression and anxiety (1, 2). According to a recent review article (3), the prevalence rate for preschool anxiety disorders was around 10–20%. Anxiety disorders were also well-recognized to have the highest prevalence rate among different types of psychiatric illness during the preschool period (3). On the other hand, the prevalence rate of depression in the preschool period was still understudied, as it could vary from 0 to 2.1% (3). In a study involved over 1,300 primary school children in Hong Kong, about 10% were reported to have major depressive disorders (4). From 2012 to 2013, The Child Assessment Service (CAS) of the Department of Health of Hong Kong diagnosed 570 new cases with anxiety disorders/problems, which accounted for 3% of the total referral cases in the Department (5). A more recent study showed the prevalence of children with anxiety disorders was 27.5% in Hong Kong (6). The prevalence and incidence of having depression and/or anxiety are expected to be even higher in children with learning difficulties and/or neurodevelopmental disorders, given they encounter more daily challenges at learning and living. For instance, the numbers of children with Attention-Deficit and/or Hyperactivity Disorder (ADHD) who are comorbid with another mental disorder were doubled compared to typically developing children (6). Given the appearance of depression and anxiety symptoms in early childhood were associated with later negative outcomes in middle childhood (7), it is essential to offer early interventions to children who are at high risk of mental illness, i.e., children with learning difficulties and/or neurodevelopmental disorders. Educational kinesiology (also called Brain Gym) could be a potential treatment to tackle this problem.

Educational kinesiology was developed by Paul Dennison and Gail Dennison in 1969 (8). It is one of the potential treatment/interventions that is popular among educators and is a training scheme that is particularly suitable for children due to its simple and easy-to-follow steps for managing stress-induced problems including anxiety. Educational kinesiology consists of a series of movements that are intended to facilitate whole-brain learning through a bottom-up approach (9). Dennison and Dennison proposed the change of brain functions happened after the change of body movements (9). Educational kinesiology program is based on the theory that the ability of learning can be blocked by the lack of individuals' coordination between the brain and the body as if they are “imbalance” (9). Thus, 26 simple movements include the Cross Crawl, the Think of an X, the Lazy Eights and the Neck Rolls, etc., are developed in this program to integrate the body with specific brain functions for easing this “imbalance.” These movements are grouped into three dimensions base on the targeted brain function: laterality, focus, and centering. These three dimensions are proposed to contribute to different areas of the brain and different functioning. *Laterality* dimension refers to the coordination between the left and right hemispheres' functioning to facilitate one's ability in reading, writing, listening, speaking, and the ability to move and think at the same time. *Focus* dimension refers to the balance of the anterior and posterior portions of the brain, facilitating comprehension skills, and improving attention. *Centering* dimension refers to the coordination of the dorsal and ventral parts of the brain. This dimension specifically targets the balance of rational thoughts and emotions (9). Educational kinesiology is different from typical aerobic or stretching exercises, although some aerobic components and stretching postures are involved. Compared to typical aerobic or stretching exercises, educational kinesiology focuses on improving body balance and muscle synergy instead of cardio and muscular endurance. Furthermore, educational kinesiology emphasizes on the use of small muscle for fine motor movements that also integrates visual and audio inputs (9). In a recent short communication article, educational kinesiology was considered as an important exercise in physiotherapy and rehabilitation that could decrease anxiety, stimulate the vestibular system for equilibrium, improve mental health and reduce stress through the activation of the brain via a bottom-up approach (10). Given the effect of educational kinesiology on stress and anxiety reduction, it is plausible that educational kinesiology might alter the stress pathway, i.e., the hypothalamic-pituitary axis (HPA). In addition, it has been well-documented that exercise could trigger the release of oxytocin from nucleus tractus solitarius, resulting in the reduction of anxiety and induction of empathy (11). It is therefore also possible that the oxytocinergic pathway could be involved in educational kinesiology-induced anxiety reduction.

Educational kinesiology is especially popular among teachers and educators. Stephenson (12) performed an internet search study using terms “Braingym” or “Brain Gym,” and “School” and found 4,290 website hits. The first 200 sites were visited to check if they are targeting teachers and educators in Australia as audiences. Thirty of which were found explicitly promoting educational kinesiology to teachers and educators. All of them included some levels of support to teachers and educators if educational kinesiology was chosen to be used in school, and most of them recommended educational kinesiology as a form of professional development.

In the past decades, different studies attempted to examine the effectiveness of educational kinesiology in various aspects. For instance, a study showed a positive effect of educational kinesiology program on learning to play musical instruments in a small group of college students (8). Another study demonstrated positive effects of educational kinesiology program on reading performance, maths performance, maladaptive behaviors, and adaptive behaviors in 30 at-risk primary school students (13). Likewise, results from another recent study showed that educational kinesiology program could improve academic performance of children aged 10–12 years old (14). In kindergartens, a recent study found that 2 weeks of daily training of the educational kinesiology could significantly improve kindergarteners' learning engagement and reading ability (15). Significant enhancement of memory ability after the educational kinesiology training was also found in another recent study among kindergarteners (16). This training was also found to be facilitating fine and gross motor development in kindergarteners (17). These studies suggest some positive effects of educational kinesiology on learning across different age groups including young children. Nevertheless, scientific evidence that supports the claimed beneficial effects of educational kinesiology remains limited, making it hardly become an evidence-based practice.

There is limited research exploring the effectiveness of educational kinesiology on the improvements of mental health, and findings from the previous studies were inconsistent. For instance, Azizah et al. (18) showed an improvement in perceived levels of psychological distress, measured by the self-reported Depression Anxiety Stress Scales (19), in elderly people after educational kinesiology intervention. Effendy et al. (20) also examined the effects of educational kinesiology program on anxiety and quality of sleep in 68 elderly people and they found that 8-week of educational kinesiology training could improve sleeping quality and reduce anxiety symptoms in the studied cohort. The anxiety levels/symptoms measured in this study was assessed via the Hamilton Rating Scale for Anxiety, which is a clinician-rated scale (21). On the other hand, Voss (22) found no effect of educational kinesiology program on the level of perceived stress in a group of school-age children, which were measured by the self-reported School Situation Survey (23). The inconsistent findings from the previous literature might be due to the fact that subjective measures were the only tool to assess emotional state, in which subjective bias is inevitable. In addition, self-reported measures were not always the best for every population. Parent-rated reports were found to be more accurate in predicting real life social approach behaviors among adults with intellectual disabilities (24). Findings from a meta-analysis conducted by Sukhodolsky et al. (25) suggested that when comparing with self-reported measures, parent-rated and clinician-rated measures were better in detecting treatment effect for anxiety among children with autism. Moreover, given the advanced development of the biotechnology, we are now able to objectively measure stress and anxiety using reliable biomarkers in a non-invasive approach.

For instance, psychological stress can be measured through the level of stress biomarkers in saliva samples, which provides accurate objective data in scientific studies. The use of stress biomarkers can avoid bias generated by the assessor and reporter in the commonly used self-reported method. This approach is well-adopted in psychological and clinical studies because of its non-bias and non-invasive nature. Given the possible involvement of the HPA and oxytocinergic pathway in educational kinesiology, this study would focus on studying the effect of educational kinesiology on cortisol and oxytocin levels.

### Cortisol

Cortisol is a stress hormone regulated by the hypothalamic-pituitary axis (HPA), which is one of the main components in the stress system (26). A meta-analysis in 2004 reviewed 208 laboratory results showing that psychological stressors increased cortisol levels, especially when the task involved uncontrollability and social evaluation (27). Furthermore, multiple studies' findings support that salivary cortisol is a useful measure of the stress response that can be used to evaluate intervention effectiveness (28–30). Being widely considered as a consistent measure of stress, studies also examined the change in salivary cortisol in preschoolers in response to stress tasks (31, 32), which further supports salivary cortisol to be a validated biomarker in measuring stress in children. In kindergartners, an increase in cortisol levels can also be captured in stressful environments (33–35), suggesting that the levels of cortisol could reflect the perceived stress among a younger age group.

### Oxytocin

Oxytocin is a neuropeptide produced in the hypothalamus. The primary biological function of oxytocin is for uterine contractions during childbirth and is necessary to produce milk (36, 37). On the other hand, oxytocin has been recognized as a love hormone due to its effects on social cognition and prosocial behaviors (38–41). More recent research revealed the association between oxytocin and mental health problems. For instance, high oxytocin levels were reported to associate with low anxiety levels in children and adolescents (42, 43). In agreement, urinary oxytocin levels were found to decrease during a social stress task in children and adolescents (44). In clinical subjects with anxiety disorder, salivary oxytocin levels were negatively correlated with anxiety symptoms, suggesting a role of oxytocin in regulating anxiety (45). In kindergarteners, Wu and Su (46) found that oxytocin levels were significantly and positively associated with prosocial behaviors in children from 3 to 5 years old, in which childhood prosocial behaviors were found to be significantly associated with a lower risk of having socio-economical difficulties in adulthood, a common adversity, in a recent population-based study (47). In addition, a longitudinal study demonstrated the association between oxytocin receptor genes polymorphism and childhood adversities starting from kindergarten years (48). These findings indicated the plausible role of oxytocin pathways in managing psychological distress in kindergarten students.

These biomarkers, on the one hand, provide objective data directly from the subject, which is not confounded by subjective bias and raters' background. On the other hand, this physiological data could help reveal the underlying physiological mechanisms of the educational kinesiology, which is important to confirm the effectiveness of the training.

Compared to other well-established group therapy methods such as music therapy and meditation that require well-trained therapists/trainers to carry out, educational kinesiology training can be carried out by professionals, trainers, educators, or even parents who have taken the basic educational kinesiology training course, which can be completed within weeks. The anti-stress and anti-anxiety effect of educational kinesiology have also been reported in different studies [see the short communication by (10)]. Yet, it remains considered as a pseudoscience because of the limited scientific evidence to support its effectiveness. To the best of our knowledge, there are no studies that investigate the underlying physiological mechanism of educational kinesiology or examine the effectiveness of educational kinesiology on mental health by measuring stress and anxiety biomarkers in children at high risk of mental health problems i.e., children with learning difficulties and/or neurodevelopmental disorders. By measuring the changes in stress and anxiety biomarkers (i.e., cortisol and oxytocin) after the intervention, we could have a better picture of the potential underlying physiological mechanisms of educational kinesiology. This study aims to investigate the changes in salivary cortisol, oxytocin, and anxiety levels after 10-week of educational kinesiology training in kindergarteners with learning difficulties or neurodevelopmental disorders. Findings from this study could provide preliminary data that informs teachers and parents the plausible effectiveness of educational kinesiology in alleviating emotional instability in kindergarteners with special needs and afford new insights into the understanding of the potential underlying physiological mechanisms of educational kinesiology.

### Hypotheses

In this study, the following hypotheses were made.

*Hypothesis 1:* The educational kinesiology intervention would reduce the levels of salivary cortisol.
*Hypothesis 2:* The educational kinesiology intervention would increase the levels of salivary oxytocin.
*Hypothesis 3:* The educational kinesiology intervention would reduce the anxiety levels of the participants.

## Methodology

### Participants

Thirty-seven subjects with special needs (age range = 3.7–6.6 years; 26 males) participated in this study, including confirmed cases of autism spectrum disorder (ASD), ADHD, developmental delay, specific learning difficulty, and at-risk cases of ASD, ADHD, conduct disorder, emotional disorders, muscle development deficits, speech delay, and developmental delay (see Table 1). They were recruited from five local mainstream nursery schools in Hong Kong. Twenty-five quota was set for each group in this study. The final sample size was determined by the number of referred students from the five targeted kindergartens. Each kindergarten served as a blocking factor in the study. Participants' age and gender were matched as much as possible between the two groups to minimize the influence of the effect of age and gender. Exclusion criteria are 1. participating in any intervention program that aims to reduce stress and anxiety; 2. unable to follow instructions (e.g., subjects with moderate to severe intellectual disability); 3. physical disabilities (e.g., deafness, upper/lower limbs dysfunction, blindness, etc.), and 4. having a history of brain injury.

**Table 1:** Descriptive statistic of participants.

**Variables**	**Control**	**Training**	***t***	***p***
	**(*N* = 21)**	**(*N* = 16)**		
Age (mean ± SD)	5.325 ± 0.805	5.109 ± 0.564	0.025	0.98
Gender (male: female)	15:6	11:5		0.86
Special needs (%)
Developmental delay	4 (10.811)	1 (2.703)		
ASD	2 (5.405)	2 (5.405)		
ADHD	0 (0)	1 (2.703)		
Specific learning difficulty	0 (0)	4 (10.811)		
At-risk cases^*^	10 (27.027)	13 (35.135)		

*ADHD, Attention Deficits and/or Hyperactivity Disorder; ASD, Autism Spectrum Disorder; SD, Standard Deviation*.

* *At-risk cases were referred by the target kindergartens including suspected ADHD, ASD, conduct disorder, emotional disorders, muscle development deficits, speech delay, and developmental delay. They were not diagnosed at the time of this study*.

### Study Design

A quasi-experimental design was adopted because of practical constraints i.e., time clash with the school scheduled activities. No potential adverse effects were anticipated in participating in the educational kinesiology training. Subject recruitment was done by Hong Kong Sheng Kung Hui Welfare Council Limited. Five kindergartens registered to the program. The kindergartens helped promote this study and recruit families who were interested in the program. Participants in three kindergartens that could meet the training schedule were assigned to the experimental group. Participants in the other two kindergartens were in the wait-list control group, and they received normal schooling without any extra interventions/trainings during the experimental period. After the completion of the project, participants in the wait-list control group were provided with the same educational kinesiology training as compensation. Participants in both groups shared the same interest in attending educational kinesiology intervention. The 10-week educational kinesiology intervention program was carried out by the same service provider. Pre-intervention assessments were carried out by the research team within 2 weeks before the start of the intervention program or the waiting period, which included a parent-rated questionnaire and saliva samples collection. Post-intervention assessments were carried out within 2 weeks after the end of the last section of the intervention or the waiting period. Assessments were done in a quiet room at the target schools on a one-to-one individual basis. Stickers were given to the participants at the end of each intervention session and after pre- and post-assessment to motivate their engagement. Written consent was obtained from the schools and parents of the participants prior to the experiment. Procedures in this study were approved by the Human Research Ethics Committee at The Education University of Hong Kong (Ref no. 2018-2019-0038).

### Educational Kinesiology Training

The educational kinesiology training was carried out by a qualified trainer who had obtained the certificate to practice educational kinesiology. The steps and details of this intervention were followed the information provided in the Brain Gym handbook (49). The intervention included 8 group-based training sections (3–4 students per group) and 2 family-based training sections for educating the participants' parents the theoretical basis of educational kinesiology and guiding them how to practice educational kinesiology with their children at home. This added up to 10 sections in total, one session per week, 60 min for each section. The intervention was carried out after classes at the kindergartens. The rundown of the group-based training section is shown in Table 2.

**Table 2:** Procedures of Brain Gym training.

	**Activities**	**Resources**
5 min	Welcome & Greeting	Hello Song
10 min	Brain Gym PACE (positive, active, clear, and energetic) movements: -Sipping water -Brain Buttons -The Cross Crawl -Hook-ups	PACE poster, water, energy ball and small equipment for learning cross crawl (self-design)
5 min	Set session goal (for specific body part)	/
20 min	Brain Gym movements include^*^: -Literality • The double doodle • Alphabet 8 s • Belly breathing • The cross crowl • Lazy 8 s -Centering • Sipping water • The thinking cap • Brain buttons • The positive points • Hook-ups (Part I & II) -Focus • The owl • The calf pump	Finger dolls, laser pointer and blank drawing cards
10 min	Brain Gym movement: Lazy 8 s. Doing a Lazy 8 s with various fun ways and tools (Claim-down period)	Worksheets, crayons
5 min	Wrap-up, appreciation and singing goodbye song	Stickers

* *Each session included 8–10 movements from the list*.

### Primary Outcome Measures: Salivary Cortisol and Oxytocin Levels

Saliva (1 mL) was collected by instructing the participant to spit out saliva directly into a measuring tube at 2–4 pm of the assessment days to avoid influence from circadian rhythm on cortisol levels (50, 51). Collected saliva samples were processed on the same day and stored at −80°C until further analysis. Cortisol and oxytocin levels were measured in saliva samples using commercially available enzyme-linked immunosorbent assay (ELISA) kits from Enzo Life Sciences (Farmingdale, NY, USA) in duplicate per sample, according to the manufacturer's instructions. The detection range of the cortisol and oxytocin kits was 156–10,000 pg/ml and 15.6–1,000 pg/ml, respectively. To avoid inter-assay variation, the pre- and post-samples of the same participant were analyzed in the same ELISA kits.

### Secondary Outcome Measures: Parent-Rated Preschool Anxiety Scale: Traditional Chinese Version (PAS-TC)

Kindergarteners' anxiety levels were measured by PAS-TC (52). It is a parent-rated questionnaire distributed and collected by the kindergartens. It has 28 items and is rated on a 4-point Likert scale [1 = never to 4 = always (53)]. The total score reflects an overall state of anxiety; the higher the scores mean the more severe the anxiety symptoms. A score equal to or higher than 34 is considered as showing elevated levels of anxiety symptoms. The Cronbach alpha for the total score in our samples was 0.875, indicating good internal consistency.

### Data Analysis

All participants provided data at the pre- and post-intervention time point. We encountered no missing data; thus, all data were used. Non-parametric tests were used for data analyses at group level in this study because of the small sample size. Between-group differences at baseline were examined by Mann-Whitney *U* test. To test our hypotheses 1 and 2, changes in salivary cortisol and oxytocin levels were calculated by subtracting the pre- from the post-data and the between-group differences in the changed values were analyzed by Mann-Whitney *U* test. Effect sizes (*r*) were calculated for each Mann-Whitney U test performed (54). In addition, Quade tests were used to examine the between-group differences in the changes in cortisol and oxytocin levels with the effect of their baseline values, gender and types of special needs being controlled (55). Briefly, the baseline cortisol and oxytocin levels as well as their changed levels were ranked. The ranked baseline levels of cortisol or oxytocin, gender, and types of special needs were set as the regressor to predict the ranked changes in cortisol or oxytocin, respectively, using linear regression models. The residuals generated from the linear regression models were then used as the dependent variables in one-way analysis of variance (ANOVA), while group was set as the independent variable. Eta-squared effect sizes were calculated for every Quade's test performed (54). For PAS-TC, those who had a score below the cut-off (score = 34) would be coded as 0, indicating normal in anxiety levels; those who had a score equal to or above the cut-off would be coded as 1, indicating an elevation of anxiety levels. The frequencies of 0 and 1 were counted in each group at baseline and post-intervention. To test our hypothesis 3, the ratio of normal to elevated anxiety subjects was compared across groups at both baseline and post-intervention using Fisher's exact tests. To calculate the change in an anxiety state, a second level of coding was performed based on the change of scores of PAS-TC i.e., post-data minus the pre-data for each participant. Participants who had an improvement in an anxiety state (e.g., the change of scores <0) were coded as 1; participants who had no changes or even deterioration in the anxiety state (e.g., the change of scores ≥0) were coded as 0. Fisher's exact tests were used to test the between-group difference in the ratio between improvement to deterioration or no changes. Phi values were calculated for the effect size. To ensure the Fisher's exact tests' results were relevant, each test was checked for no more than 20% of cells with expected frequencies <5 and no cells have expected frequency <1 (56). To examine the linkage between the physiological measures and the psychological measure, the change of neurohormone levels were compared between participants with the change in anxiety levels indicated from PAS-TC regardless of the training conditions. Participants were first categorized based on the cut-off scores from the PAS-TC. Then, participants can be classified into two groups based on the categorical change of the PAS-TC between pre-and post-time points: 1. Improved anxiety levels; 2. No changes or even increased in anxiety levels. Mann-Whitney *U* tests were performed to compare the change of neurohormone levels between these two groups. Statistical analyses were done using SPSS (IBM, version 24.0). A *p*-value < 0.05 is considered statistically significant.

## Results

The descriptive statistics of the participants are summarized in Table 1. Sixteen participants joined the intervention group, while the remaining 21 participants joined the control group. All 37 of the participants agreed to participate once they were informed with the study details, and all completed all stages of this experiment. Three dependent variables were analyzed at baseline as well as the changes between pre- and post-intervention, including the PAS-TC, cortisol and oxytocin levels. Summary of the hormonal results are presented in Table 3.

**Table 3:** Group differences on changes in salivary cortisol and oxytocin levels.

**Dependent variables**	**Groups**	***N***	**Mann-whitney** ***U***								Quade test^*^		
			**Baseline mean ± SE (pg/mL)**	***U***	***p***	***r***	**Mean of change ± SE (pg/mL)**	***U***	***p***	***r***	***F***	***p***	**eta^**2**^**
Cortisol	Control	21	3042.231 ± 181.683	65	**0.002**	0.519	−118.681 ± 177.892	99	**0.034**	0.348	0.306	0.584	0.009
	Training	16	4502.692 ± 651.226				−1,200.519 ± 607.781						
Oxytocin	Control	21	44.619 ± 3.611	37	**<0.001**	0.660	3.186 ± 4.375	102	**0.043**	0.333	4.747	**0.036**	0.119
	Training	16	140.626 ± 25.629				78.701 ± 51.885						

* *Quade Test: Between group differences in the change of cortisol/oxytocin levels, the effect of baseline levels, gender, and types of special needs were controlled. Statistically significant differences were bold*.

### Hypothesis 1: Effect of Educational Kinesiology on Cortisol Levels

**Figure 1:**
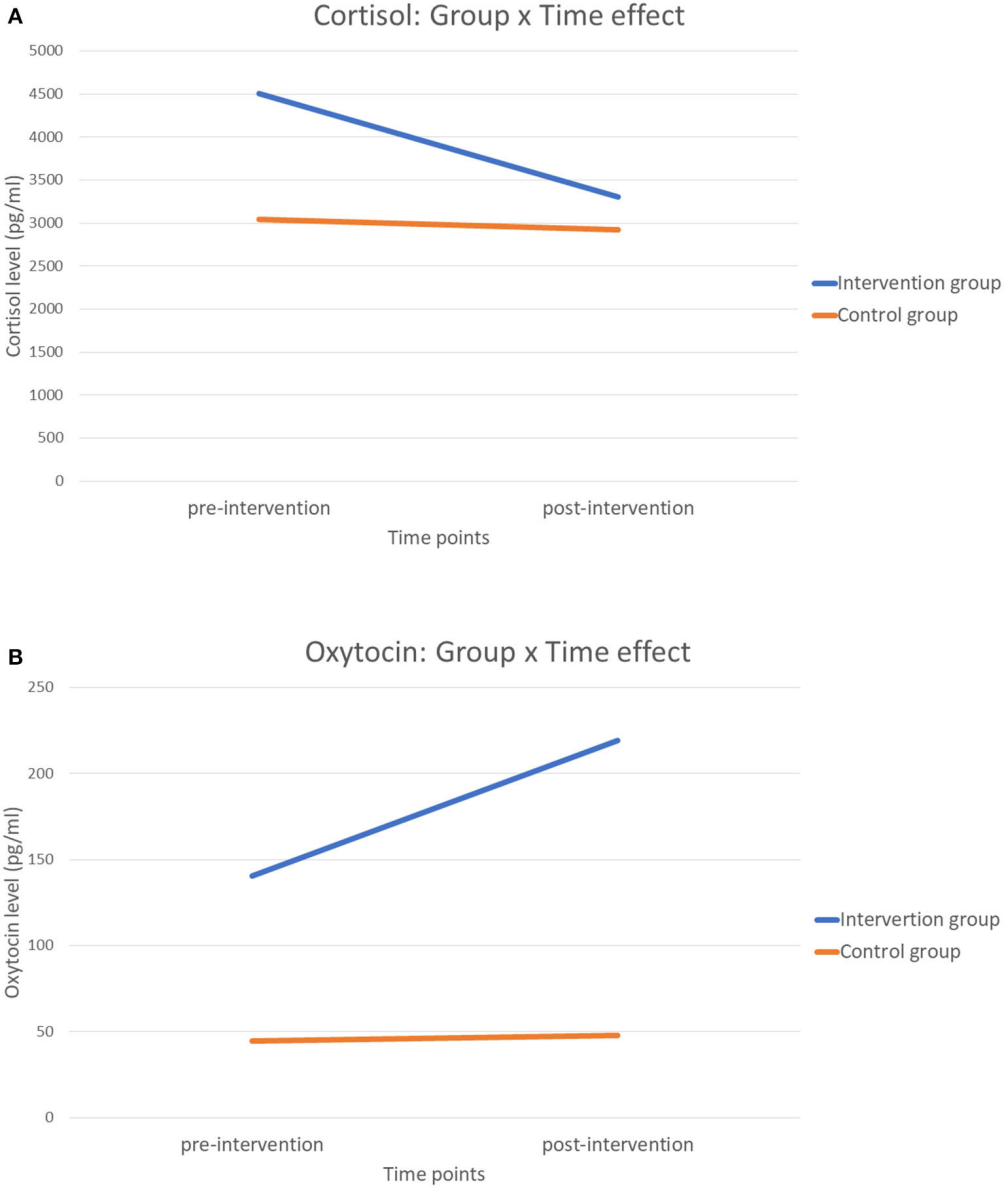
**(A)** The group by time effect in cortisol levels. **(B)** The group by time effect in oxytocin levels.

The baseline cortisol levels were found significantly higher in the training group (4,502.692 ± 651.226 pg/ml) compared with the control group (3,042.231 ± 181.683 pg/ml, *U* = 65, *p* = 0.002), with a medium effect size (*r* = 0.519). Significant between-group differences in the change of cortisol levels were observed (*U* = 99, *p* = 0.034), in which a larger reduction in the intervention group (−1,200.519 ± 607.781 pg/ml) than the control group (−118.681 ± 177.892 pg/ml) was observed (*r* = 0.348). However, the group difference became non-significant after controlled for the baseline values, gender, and types of special needs [*F*_(1, 35)_ = 0.306, *p* = 0.584, eta^2^ = 0.009, Table 3]. The group by time effect in cortisol levels was shown in Figure 1A.

### Hypothesis 2: Effect of Educational Kinesiology on Oxytocin Levels

The baseline oxytocin levels were significantly higher in the intervention group (140.626 ± 25.629 pg/ml) compared with the control group (44.619 ± 3.611 pg/ml, *U* = 37, *p* < 0.001) with a medium effect size (*r* = 0.66). The between-group differences in the change of oxytocin levels were significant (*U* = 102, *p* = 0.043, *r* = 0.66), in which the intervention group showed a larger increase (78.701 ± 51.885 pg/ml) than the control group (3.186 ± 4.375 pg/ml). The between-group difference in the change of oxytocin levels remained significant after controlled for the effect of baseline oxytocin values, gender, and the types of special needs [*F*_(1, 35)_ = 4.747, *p* = 0.036, eta^2^ = 0.119, Table 3]. The group by time effect in oxytocin levels was shown in Figure 1B.

### Hypothesis 3: Effect of Educational Kinesiology on Anxiety

No significant between-group differences in the ratio of normal anxiety level to elevated anxiety level were observed at baseline (*p* = 0.999). After training, there was a trend level difference in the ratio of normal anxiety level to elevated anxiety level between the intervention and the control group (*p* = 0.093), in which a larger ratio of normal to elevated anxiety level was observed in the intervention group compared with the control (ϕ = −0.321, *p* = 0.093). To further investigate the between group differences in individual improvement of anxiety level, the changed scores were calculated. An improvement in an anxiety state (e.g., the change of scores <0) was coded as 1; no changes or deterioration in the anxiety state (e.g., the change of scores ≥0) were coded as 0. There was a significant difference in the ratio of improvements to deterioration/no change between the two groups (*p* = 0.048) with a medium effect size (ϕ = 0.344, *p* = 0.048). In the intervention group, there were fewer cases who showed deterioration/no changes in their anxiety levels measured by PAS-TC (*n* = 3) compared with the control group (*n* = 11). On the other hand, the number of participants who showed improvement in the anxiety levels after the intervention/waiting period was slightly more in the intervention group (*n* = 13) than the control group (*n* = 10) (see Table 4).

**Table 4:** Fisher's exact tests for the PAS-TC.

	**Conditions**	**Control**	**Training**	***p***	**ϕ**
		**(*N* = 21)**	**(*N* = 16)**		
Baseline	Normal : elevation of anxiety	9:12	7:9	0.999	−0.009
Post-intervention	Normal : elevation of anxiety	9:12	12:4	0.093	−0.321
Change	Improvements: deterioration/no change	10:11	13:3	**0.048**	0.344

*0 cells (0.0%) have expected count <5, and no cell has expected frequency <1 for all tests. Statistically significant differences were bold*.

**Figure 2:**
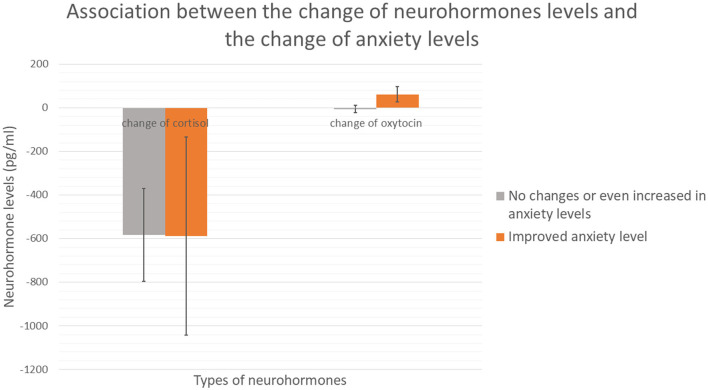
Association between the change of neurohormones and the change of anxiety levels.

In terms of the difference on the change of neurohormone levels between participants with the change in anxiety levels indicated from PAS-TC regardless the training conditions, both the changes of cortisol levels and the changes of oxytocin levels were compared between participants. There was no significant group difference in the change of cortisol levels (*U* = 129, *p* = 0.316, *r* = 0.165). As for the change in oxytocin levels, there was a trend level difference between groups (*U* = 104, *p* = 0.074, *r* = 0.293). Figure 2 demonstrated the association between the change of neurohormones level and the change of anxiety levels.

## Discussion

This is the first study that investigates changes in cortisol and oxytocin levels and improvement in anxiety after educational kinesiology in kindergarteners with special needs. Our findings demonstrated that the 10-week educational kinesiology intervention could increase salivary oxytocin levels and suppress the deterioration of anxiety levels in kindergarteners with special needs, which robustly support our second hypothesis. We also observed a significant greater reduction in salivary cortisol levels in the intervention group compared with the control. However, such difference became non-significant after controlled for the effect of the baseline cortisol levels, gender and types of special needs. In this study, we cannot draw a definite conclusion on the first hypothesis due to the small sample size and the inconsistent findings after controlled for the effect of the baseline cortisol levels, gender and types of special needs.

Educational kinesiology has been reported to reduce stress in elderly people (18). Our findings provide preliminary evidence on the stress reduction effect of educational kinesiology by demonstrating a greater reduction of salivary cortisol levels after 10-week of educational kinesiology intervention than the control in kindergarteners with special needs. The significant difference in cortisol levels between groups before controlling for baseline indicated the trend of change in cortisol as the effect of the training. However, it became non-significant after controlling for the effect of baseline cortisol levels, gender, and types of special needs. Such discrepancy could be explained by the fact that there was a significant difference in baseline cortisol levels, which could not be eliminated due to the non-randomized design and plausible site effects in this study. In addition, the effect of circadian rhythm on cortisol levels could also impact our findings. Albeit the cortisol levels in both groups were still within a normal range (28), fluctuations in the cortisol levels could be possible even when the time window of saliva collection in this study was limited to 2–4 pm (50, 51, 57). This kind of individual difference is even more obvious when a small sample is included. As the sample size is one of the limitations in this study, the interpretation and generalizability of the cortisol findings were limited. Nevertheless, we cannot prove that educational kinesiology training could modulate cortisol levels based on our data. Given that cortisol is a stress hormone regulated by one of the main components in the stress system, HPA (26), it remains unclear whether educational kinesiology could reduce stress and anxiety via modulation of the HPA system. Future studies will be needed to examine this aspect with a larger sample.

Oxytocin has been associated with stress and anxiety. It was highly correlated with cortisol during a social stress task, and its levels in saliva were negatively associated with anxiety and insecurity (42). Similar findings that negative association between higher salivary oxytocin levels and lower anxiety levels were reported in hospitalized children (43). In youths with anxiety disorder, salivary oxytocin levels were also found to negatively correlate with anxiety symptoms (45). These findings suggest the anti-anxiety role of oxytocin. A previous study demonstrated that 8-week educational kinesiology intervention significantly decreased anxiety levels in 68 elderly people (18). We observed significant group difference in the change of oxytocin levels with or without controlled for the effect of baseline oxytocin levels, gender, and types of special needs, in which greater increases in oxytocin levels were observed in the intervention group compared with the control. In addition, we found a trend level difference in the change of oxytocin levels between subjects with and without improvement in anxiety levels regardless of the training condition. This data further suggests a plausible linkage between oxytocin and anxiety. Oxytocin is known as a love hormone that plays an important role in social functioning and bonding with significant others. Physiologically, oxytocin modulates cell viability, synaptic and structural plasticity in neurons (58–60). In pre-clinical studies, oxytocin was found to reduce anxiety-like behaviors through its neuromodulatory role on the amygdala, a core hub in the brain that mediates fear and anxiety (61). A recent study further reported the importance of the oxytocinergic projections from the paraventricular nucleus of the hypothalamus to the central amygdala in the discrimination of both positively and negatively valenced emotional states (62). Our findings of increased salivary oxytocin levels and improvement in anxiety level after 10-week educational kinesiology intervention supports the potential anti-anxiety effect of educational kinesiology in kindergarteners with special needs via the oxytocin pathway. In addition, our findings suggest that salivary oxytocin could be a sensitive marker that informs the treatment efficacy in reducing anxiety in this specific cohort. Nevertheless, contrasting evidence was also found from the literature, indicating that oxytocin increased anxiety to unpredictable threats (63). Future studies should combine biological and neuroimaging data to further investigate this aspect.

In terms of the subjective measure, there was a significant difference in the ratio of improvements to deterioration or no change of the PAS-TC scores between the groups. Although the numbers of improvements were similar for both groups, our findings showed that educational kinesiology reduced the deterioration of anxious and stressed emotions during early childhood. The trend level between-group difference in the ratio of normal to elevated anxiety levels further indicates the plausible anti-anxiety effect of educational kinesiology, which partially supports our third hypothesis. Taken together, we speculate that through the alteration of oxytocin pathway, anti-anxiety effects may be achieved after the educational kinesiology intervention (26).

The strength of the current study is the application of both psychometrics and standardized and validated biological markers in describing stress and anxiety. Previous studies examining the effectiveness of educational kinesiology adopted only subjective measures (18, 20, 22), in which subjective bias is inevitable. Our findings indicate that salivary oxytocin but not cortisol levels could be a sensitive marker for capturing the treatment effect of educational kinesiology on the improvement of anxiety symptoms in kindergarteners with special needs.

### Limitations

There are several limitations in this study. First, a quasi-experimental design was adopted because of the practical constraint. Subject bias and influence from the experiment sites cannot be avoided, which limits the interpretation of our findings. Second, we included subjects with special needs in general but not focused on a specific disorder. The types of disorder and/or the severities can be potential confounding variables affecting the training effect. Nevertheless, our main findings were the same after controlling for the effect of types of special needs in the Quade tests, indicating that the influence of types of special needs on the training effect could be minimum in our samples. Future studies are recommended to focus on a specific type of special need or disorder to control for the influence. Last, the sample size was relatively small in this study, the generalizability was therefore limited. In addition, the small sample size could result in a higher false positive and false negative rate. Although our findings in the change of oxytocin levels after training were robust, future studies with a larger sample and adopt a randomized controlled setting are strongly recommended to confirm our findings and investigate other potential changes.

### Conclusion

In conclusion, our findings indicate potential anti-anxiety effects of educational kinesiology in kindergarteners with special needs, plausibly via the elevation of oxytocin levels. These findings suggest that the oxytocin pathway could be a potential underlying mechanism of educational kinesiology, which requires further study to confirm. In addition, salivary oxytocin appears to be a sensitive marker to inform the treatment efficacy of educational kinesiology. Given educational kinesiology is safe and easy to perform, it is recommended for clinicians and educators to adopt it as a training option for helping children with stress and anxiety problems. Nevertheless, more high-quality empirical studies are desired to investigate the physiological and neural mechanisms of educational kinesiology before it can be developed into an evidence-based practice.

## Data Availability Statement

The original contributions presented in the study are included in the article/supplementary material, further inquiries can be directed to the corresponding author/s.

## Ethics Statement

The studies involving human participants were reviewed and approved by Human Research Ethics Committee at The Education University of Hong Kong (Ref no. 2018-2019-0038). Written informed consent to participate in this study was provided by the participants' legal guardian/next of kin.

## Author Contributions

AT: collection of data, data analysis and interpretation, and manuscript writing. WL: conceptualized and designed the research, provision of the study materials and participants, collection and assembly of data, data analysis and interpretation, and manuscript writing. Both authors contributed to the article and approved the submitted version.

## Funding

This study was supported by the funding provided by Central Reserve Allocation Committee at The Education University of Hong Kong (awarded to WL).

## Conflict of Interest

The authors declare that the research was conducted in the absence of any commercial or financial relationships that could be construed as a potential conflict of interest.

## Publisher's Note

All claims expressed in this article are solely those of the authors and do not necessarily represent those of their affiliated organizations, or those of the publisher, the editors and the reviewers. Any product that may be evaluated in this article, or claim that may be made by its manufacturer, is not guaranteed or endorsed by the publisher.

## Abbreviations

ADHD: Attention-Deficit and/or Hyperactivity Disorder
ANOVA: Analysis of Variance
ASD: Autism Spectrum Disorder
CAS: The Child Assessment Service
CONSORT: The Consolidated Standards of Reporting Trials
ELISA: Enzyme-linked Immunosorbent Assay
HPA: The Hypothalamic-pituitary Axis
PACE, Positive, Active: Clear and Energetic
PAS-TC: Parent-rated Preschool Anxiety Scale: Traditional Chinese Version
SD: Standard Deviation.
